# Proteinuria Indicates Decreased Normal Glomeruli in ANCA-Associated Glomerulonephritis Independent of Systemic Disease Activity

**DOI:** 10.3390/jcm10071538

**Published:** 2021-04-06

**Authors:** Désirée Tampe, Peter Korsten, Philipp Ströbel, Samy Hakroush, Björn Tampe

**Affiliations:** 1 Department of Nephrology and Rheumatology, University Medical Center Göttingen, 37075 Göttingen, Germany; desiree.tampe@med.uni-goettingen.de (D.T.); peter.korsten@med.uni-goettingen.de (P.K.); 2 Institute of Pathology, University Medical Center Göttingen, 37075 Göttingen, Germany; philipp.stroebel@med.uni-goettingen.de (P.S.); samy.hakroush@med.uni-goettingen.de (S.H.)

**Keywords:** small vessel vasculitis, ANCA glomerulonephritis, proteinuria

## Abstract

Background: Renal involvement is a common and severe complication of antineutrophil cytoplasmic antibody (ANCA)-associated vasculitis (AAV), potentially resulting in a pauci-immune necrotizing and crescentic ANCA glomerulonephritis (GN) with acute kidney injury (AKI), end-stage renal disease (ESRD) or death. There is recent evidence that the degree of proteinuria at diagnosis is associated with long-term renal outcome in ANCA GN. Therefore, we here aimed to systematically describe the association between proteinuria and clinicopathological characteristics in 53 renal biopsies with ANCA GN and corresponding urinary samples at admission. Methods: A total number of 53 urinary samples at admission and corresponding renal biopsies with confirmed renal involvement of AAV were retrospectively included from 2015 to 2021 in a single-center study. Results: Proteinuria correlated with myeloperoxidase (MPO) subtype, diagnosis of microscopic polyangiitis (MPA) and severe deterioration of kidney function. Proteinuria was most prominent in sclerotic class ANCA GN and ANCA renal risk score (ARRS) high risk attributed to nonselective proteinuria, including both glomerular and tubular proteinuria. Finally, there was no association between proteinuria and systemic disease activity, suggesting that proteinuria reflected specific renal involvement in AAV rather that systemic disease activity. Conclusions: In conclusion, proteinuria correlated with distinct clinicopathological characteristics in ANCA GN, mostly attributed to a reduced fraction of normal glomeruli. Furthermore, proteinuria in ANCA GN reflected specific renal involvement in AAV rather than systemic disease activity. Therefore, urinary findings could further improve our understanding of mechanisms promoting kidney injury and progression of ANCA GN.

## 1. Introduction

Antineutrophil cytoplasmic antibody (ANCA)-associated vasculitis (AAV) is a small vessel vasculitis according to the 2012 revised Chapel Hill Consensus Conference Nomenclature of Vasculitides, most frequently presenting as microscopic polyangiitis (MPA) or granulomatosis with polyangiitis (GPA) [1,2]. The most severe form of AAV requiring intensive care presents with severe renal and pulmonary involvement, contributing to mortality [3]. Renal involvement is a common complication of AAV, potentially resulting in a pauci-immune necrotizing and crescentic ANCA glomerulonephritis (GN) with acute kidney injury (AKI), end-stage renal disease (ESRD) or death [2]. Clinicopathologic studies of the European Vasculitis Study Group (EUVAS) could demonstrate that the percentage of normal glomeruli, global glomerular sclerosis and the degree of interstitial fibrosis/tubular atrophy (IF/TA) are important parameters related to renal outcome in necrotizing and crescentic GN [4,5,6,7]. Derived from these studies, histopathological subgrouping into four classes (focal, crescentic, mixed, and sclerotic) as defined by Berden et al. was shown to predict long-term renal survival rates poorest in the sclerotic class (sclerotic glomeruli above 50%) [8]. These results were confirmed in multiple independent studies over the past years [9,10,11,12,13,14,15,16,17,18,19,20,21,22,23,24,25]. However, multivariable analyses demonstrated no improvement of outcome prediction in most of these studies, mainly attributed to no outcome difference in the crescentic and mixed classes [17,18,19,20,21,22,23,24,25,26,27,28]. Therefore, Brix et al. suggested the ANCA renal risk score (ARRS), by incorporation of combined IF/TA to the percentage of normal glomeruli and baseline glomerular filtration rate (GFR), to predict ESRD in patients with AAV [29].

The aforementioned previous studies mainly focused on deterioration of kidney function in combination with histopathological findings in ANCA GN. However, there is recent evidence that the degree of proteinuria at diagnosis is associated with long-term renal outcome in ANCA GN [25,30,31]. At disease manifestation, the majority of patients with ANCA GN have proteinuria with variable amounts [18]. Previously, we also reported that proteinuria is observed in a considerable subset of ANCA GN and associated with acute deterioration of kidney function at disease onset, implicating an association with active lesions and ANCA manifestation in the kidney [32]. This is confirmed by recent findings that proteinuria associates with established ANCA scoring systems showed the lowest proteinuria in biopsies categorized as focal class, followed by mixed and crescentic class ANCA GN [33]. However, correlation of proteinuria and its subclassification with regard to histopathological findings in ANCA GN has not been systematically described yet. Therefore, we here aimed to systematically describe the association between proteinuria and clinicopathological characteristics in 53 renal biopsies with ANCA GN and corresponding urinary samples at admission.

## 2. Materials and Methods

### 2.1. Study Population

A total number of 53 renal biopsies with confirmed renal involvement of AAV and 53 corresponding urinary samples primarily admitted to the Department of Nephrology and Rheumatology, University Medical Center Göttingen, Germany were retrospectively included from 2015 to 2021; the patient cohort was in part previously described [32]. The study was conducted according to the guidelines of the Declaration of Helsinki and approved by the Ethics Committee of University Medical Center Göttingen, Germany. Medical records were used to obtain data on age, sex, diagnosis (MPA or GPA) and laboratory results. The estimated glomerular filtration rate (GFR) was calculated using the Chronic Kidney Disease Epidemiology Collaboration (CKD-EPI) equation [34]. The worst measurement during the initial course of the disease was used to determine the severity of kidney injury. At admission, the Birmingham Vasculitis Activity Score (BVAS) version 3 was calculated as described previously [35]. The BVAS is assessed on a scale of 0 to 63, with a score of 0 indicating the absence of disease activity and higher scores indicating active disease.

### 2.2. Urinary Analysis

Levels of total proteinuria, urinary albumin, immunoglobulin G (IgG), α_1_-microglobulin and α_2_-macroglobulin were normalized to urinary creatinine concentration to control for variations in urine flow rate [36]. Leukocyturia and hematuria per high-power field (HPF) were semiquantitatively scored negative (0), 2–4/HPF (1), 5–9/HPF (2), 10–20/HPF (3) and >20/HPF (4). Acanthocytes were scored for presence/absence.

### 2.3. Renal Histopathology

A pathologist (SH) evaluated all biopsies, independently validated by a second renal pathologist (PS) blinded to clinical data collection and analysis. Within a renal biopsy specimen, each glomerulus was scored separately for the presence of necrosis, crescents and global sclerosis. Consequently, the percentage of glomeruli with any of these features was calculated as a fraction of the total number of glomeruli in each renal biopsy. Based on these scorings, histopathological subgroupings according to Berden et al. (focal, crescentic, mixed or sclerotic class) and ARRS according to Brix et al. (low, medium or high risk) were performed [8,29]. Apart from these categories, the degree of interstitial fibrosis and tubular atrophy (IF/TA) was quantified. Renal pathologists were blinded to clinical data collection and analysis.

### 2.4. Statistical Methods

Variables were tested for normal distribution using the Shapiro–Wilk test. Non-normally distributed continuous variables are expressed as median and interquartile range (IQR), categorical variables are presented as frequency and percentage. Statistical comparisons were not formally powered or prespecified. For group comparisons, the Mann–Whitney U-test was used to determine differences in medians. Nonparametric between-group comparisons were performed with Pearson’s Chi-square test. Data analyses were performed with GraphPad Prism (version 8.4.3 for MacOS, GraphPad Software, San Diego, California, U.S.). Multiple regression analyses were performed using IBM SPSS Statistics (version 27 for MacOS, IBM Corporation, Armonk, NY, U.S.). A probability (p) value of <0.05 was considered statistically significant.

## 3. Results

### 3.1. Proteinuric Findings in ANCA GN

**Figure 1:**
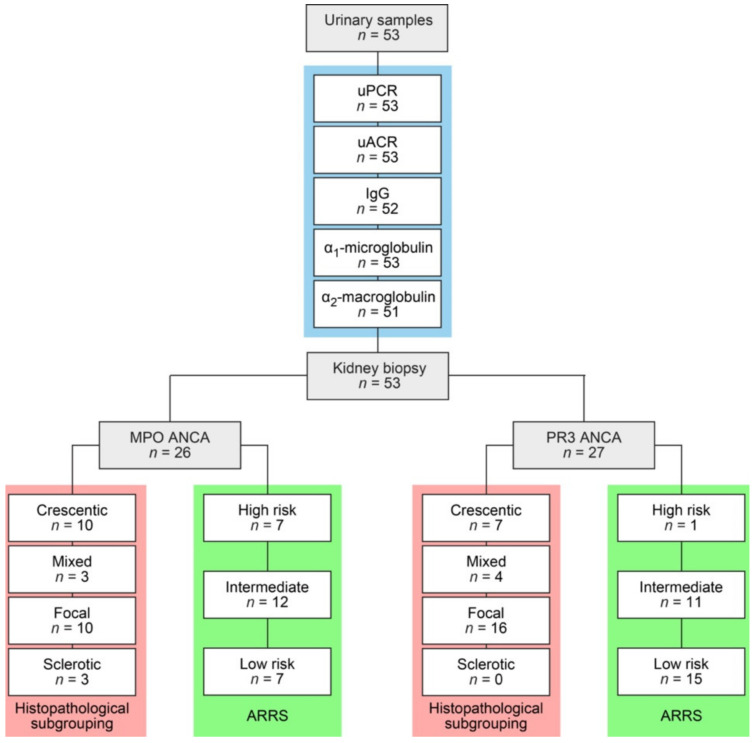
Total patient cohort of ANCA glomerulonephritis (GN). STROBE flow chart of patient disposition. Abbreviations: ANCA, antineutrophil cytoplasmic antibodies; ARRS, ANCA renal risk score; MPO, myeloperoxidase; PR3, proteinase 3; STROBE, Strengthening the Reporting of Observational Studies in Epidemiology; uACR, urinary albumin-to-creatinine ratio; uPCR, urinary protein-to-creatinine ratio.

A total number of 53 renal biopsies with confirmed renal involvement of AAV were identified and retrospectively included from 2015 to 2021. Histopathological subgroupings revealed 17/53 (32.1%) crescentic, 7/53 (13.2%) mixed, 26/53 (49.1%) focal and 3/53 (5.7%) sclerotic class ANCA GN [8]. ARRS was high in 8/53 (15.1%), intermediate in 23/53 (43.4%) and low risk class ANCA GN in 22/53 (41.5%) cases (Figure 1) [29].

The baseline characteristics of the entire cohort are shown in Table 1. In this cohort, 23/53 (43.4) were female and all were Caucasian. The median (IQR) age at diagnosis was 65 (54.5–74.5) years. Disease onset was 18 (7–46) days before admission, kidney biopsy was performed 6 (3–9.5) days after admission to confirm renal involvement of AAV. There were 26/53 (49.1%) positive for myeloperoxidase (MPO) and 27/53 (50.9%) positive for proteinase 3 (PR3) ANCA. There were 27/53 (50.9%) patients categorized as MPA and the remainder as GPA. The majority of them (84.9%) had a new diagnosis of AAV. There were 44/53 patients (83%) with extrarenal manifestation of AAV (31 with lung, 9 with sinus, 12 with joint, 4 with ear, 3 with eye, 6 with peripheral nerve and 8 with skin involvement); 7/53 (13.2%) had alveolar hemorrhage. The worst median (IQR) eGFR at disease onset was 19 (9.7–50.2) mL/min/1.73 m^2^, and 16 required dialysis within 30 days after admission. Renal involvement of AAV revealed variable protein-to-creatinine ratios (uPCR) ranging from low-range to nephrotic syndromes; however, most patients presented with subnephrotic proteinuria. This was attributed to nonselective proteinuria, including urinary albumin (uACR), IgG, α_1_-microglobulin and α_2_-macroglobulin, confirming that ANCA GN can present with glomerular, tubular or overflow proteinuria [37].

**Figure 2:**
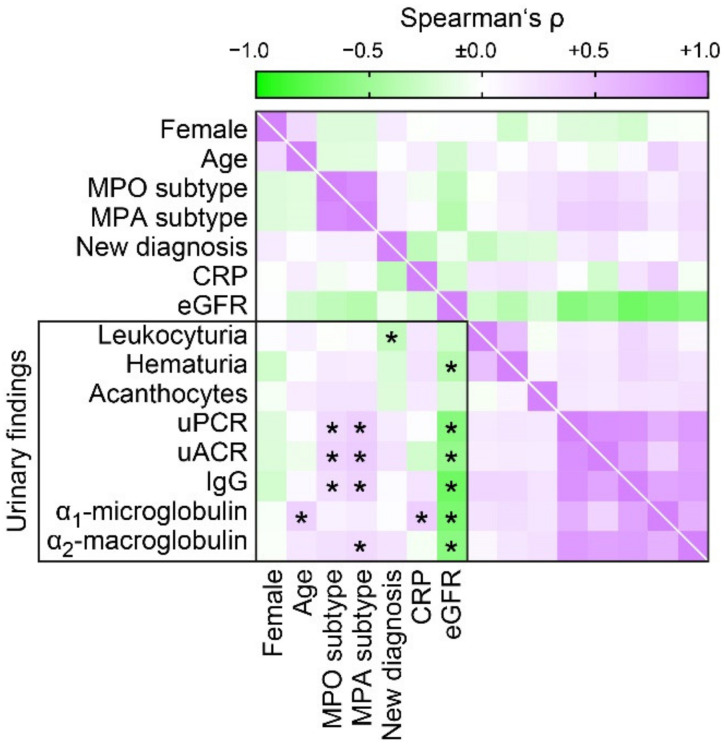
Proteinuric findings in correlation with clinical and laboratory findings in ANCA GN. Association between proteinuria, clinical and laboratory findings is shown by heatmap reflecting mean values of Spearman’s *ρ*, asterisks (*) indicate *p* < 0.05. Abbreviations: ANCA, antineutrophil cytoplasmic antibodies; CRP, C-reactive protein; eGFR, estimated glomerular filtration rate (CKD-EPI); GN, glomerulonephritis; IgG, immunoglobulin G; MPA, microscopic polyangiitis; MPO, myeloperoxidase; uACR, urinary albumin-to-creatinine ratio; uPCR, urinary protein-to-creatinine ratio.

Association with clinical and laboratory findings revealed that uPCR correlated with MPO subtype, diagnosis of MPA and severe deterioration of kidney function assessed by eGFR loss (Figure 2), in line with our previous observations [32]. Proteinuria subclassification established that higher levels of proteinuria in MPO subtype was attributed to increased uACR and urinary IgG levels (Figure 2), reflecting nonselective glomerular proteinuria. Association between severe deterioration of kidney function was observed for uACR, IgG, α_1_-microglobulin and α_1_-macroglobulin (Figure 2). Interestingly, increased levels of C-reactive protein (CRP) were specifically associated with α_1_-microglobulin (Figure 2), confirming previous observations that urinary α_1_-microglobulin reflects systemic inflammation [38]. In addition, leukocyturia was more frequently observed in patients with relapsing ANCA GN (Figure 2). In summary, ANCA GN presents with subnephrotic proteinuria in most cases, including glomerular and/or tubular proteinuria. Furthermore, proteinuria correlated with more severe deterioration of kidney function in ANCA GN. Therefore, we next aimed to correlate proteinuric findings in association with histopathological findings in ANCA GN.

### 3.2. Proteinuric Findings in Correlation with Histopathological Findings in ANCA GN

By applying scoring of ANCA GN according to Berden et al., highest levels of proteinuria (uPCR) were observed in sclerotic and lowest in focal class ANCA GN (Table 2), in line with previous observations [8,33]. Comparable results were also observed for proteinuria subclassification; uACR, IgG, α_1_-microglobulin and α_2_-macroglobulin were most prominent in sclerotic and lowest in focal class ANCA GN (Table 2). Categorization of ANCA GN in ARRS revealed that increased risk class was associated with higher levels of uPCR, equally reflected by proteinuria subclassification in uACR, IgG, α_1_-microglobulin and α_2_-macroglobulin (Table 3).

**Figure 3:**
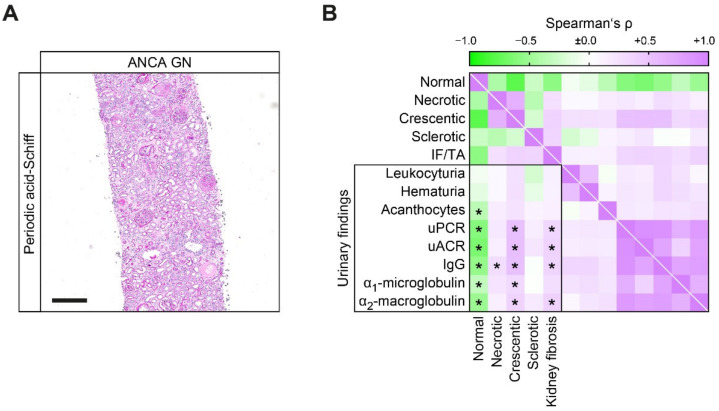
Proteinuric findings in correlation with distinct histopathological findings in ANCA GN. (**A**) Representative photomicrograph of ANCA GN stained with periodic acid-Schiff (scale bar: 300 μm). (**B**) Association between proteinuria and histopathological findings is shown by heatmap reflecting mean values of Spearman’s *ρ*, asterisks (*) indicate *p* < 0.05. Abbreviations: ANCA, antineutrophil cytoplasmic antibodies; GN, glomerulonephritis; IF/TA, interstitial fibrosis/tubular atrophy; IgG, immunoglobulin G; uACR, urinary albumin-to-creatinine ratio; uPCR, urinary protein-to-creatinine ratio.

Based on these observations that categorization of ANCA GN into established histopathological scoring systems into sclerotic class ANCA GN and ARRS high risk were associated with highest levels of proteinuria attributed to nonselective proteinuria, we next correlated proteinuric findings in association with distinct histopathological lesions in ANCA GN. Nonselective proteinuria, including uPCR, uACR, urinary IgG, α_1_-microglobulin and α_2_-macroglobulin was associated with a decreased fraction of normal glomeruli attributed to crescentic ANCA GN (Figure 3A,B). Furthermore, IF/TA also correlated with uPCR, uACR, urinary IgG and α_2_-macroglobulin, but not α_1_-microglobulin (Figure 3B).

Multiple regression analyses revealed a stronger correlation between glomerular proteinuria and a decreased fraction of normal glomeruli as compared to other glomerular lesions or IF/TA in ANCA GN (Table 4), consistent with the concept that each glomerular lesion contributing to a decreased fraction of normal glomeruli needs to be considered [29]. In summary, proteinuria is an independent indicator of decreased normal glomeruli in ANCA GN. Because proteinuria and decreased fraction of normal glomeruli could reflect both specific renal involvement and systemic severity of AAV, we next determined extrarenal manifestation of AAV in association with proteinuria.

### 3.3. Proteinuric Findings in Correlation with Extrarenal Manifestations of AAV

**Figure 4:**
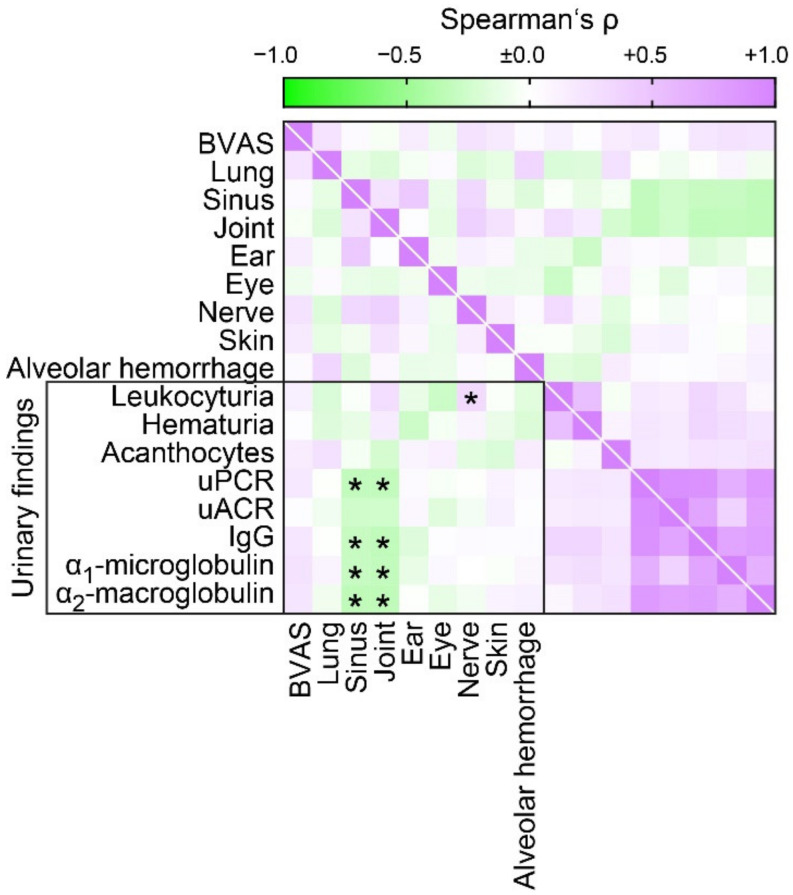
Proteinuric findings in correlation with extrarenal manifestations in AAV. Association between proteinuria and extrarenal manifestations is shown by heatmap reflecting mean values of Spearman’s *ρ*, asterisks (*) indicate *p* < 0.05. Abbreviations: ANCA, antineutrophil cytoplasmic antibodies; BVAS, Birmingham Vasculitis Activity Score; GN, glomerulonephritis; IgG, immunoglobulin G; uACR, urinary albumin-to-creatinine ratio; uPCR, urinary protein-to-creatinine ratio.

We next examined the correlation between proteinuria and extrarenal manifestation of AAV. We observed no association between proteinuria and systemic disease activity assessed by the BVAS despite previously observed correlation between proteinuria with more severe deterioration of kidney function in ANCA GN (Figure 4), implicating that proteinuria associates with decreased extrarenal manifestations of AAV. By systematic analysis of lung, sinus, joint, ear, eye, peripheral nerve, skin involvement or alveolar hemorrhage, we confirmed less involvement of sinus and joint in association with proteinuria (Figure 4). In summary, these observations suggested that proteinuria reflected specific renal involvement in AAV rather that systemic disease activity.

## 4. Discussion

We here aimed to systematically describe the correlation between urinary findings and clinicopathological characteristics in ANCA GN. Proteinuria correlated with MPO subtype, diagnosis of MPA and severe deterioration of kidney function, in line with our previous observations [32]. Proteinuria subclassification established that higher levels of proteinuria in MPO subtype were attributed to nonselective glomerular proteinuria. At disease manifestation, renal involvement of AAV can either present with active lesions including glomerular crescents and necrosis, or with chronic lesions including global glomerular sclerosis. The pathologic activity and chronicity of ANCA GN can be classified by histopathological subgrouping (crescentic, mixed, focal and sclerotic) or ARRS (high, intermediate and low risk) [8,29]. Aforementioned previous studies have mainly focused on deterioration of kidney function in combination with histopathological findings in ANCA GN. However, there is recent evidence that the degree of proteinuria at diagnosis is associated with long-term renal outcome in ANCA GN [25,30,31]. Proteinuria is the hallmark of GN and the most important predictor of outcome, including diabetes-related and idiopathic glomerular kidney diseases [39,40,41,42,43,44,45,46,47]. We here show that categorization of ANCA GN into established histopathological scoring systems revealed that sclerotic class ANCA GN and ARRS high risk were associated with highest levels of proteinuria attributed to nonselective proteinuria, including both glomerular and tubular proteinuria. In contrast, lowest proteinuria was observed in focal class ANCA GN, in line with recent findings that proteinuria associates with established ANCA scoring systems showing lowest proteinuria in biopsies categorized as focal class, followed by mixed and crescentic class ANCA GN [33]. Multivariate regression analyses revealed that only decreased fraction of normal glomeruli independently associated with proteinuria. It has previously been proposed that damaged glomeruli have the capability to recover and active lesions may be reversible in principle, at least to a certain extent [5,30]. In addition, the fraction of normal glomeruli (without scarring, crescents or necrosis within the tuft) was the strongest independent predictor of death-censored ESRD in ANCA GN [29]. Therefore, each glomerular lesion needs to be considered, emphasizing the importance of normal glomeruli and an early diagnosis. Studies of repeat biopsies strengthen this hypothesis since previous studies have demonstrated the progression of active into chronic lesions in ANCA GN over time [30,48]. This could in part explain the variable outcomes of patients that are classified as crescentic and mixed in the validation of the classification [9,14,22,24].

Finally, there was no association between proteinuria and systemic disease activity assessed by the BVAS despite previously observed correlation between proteinuria with more severe deterioration of kidney function in ANCA GN. Since the BVAS also includes deterioration of kidney function, these results implicate that proteinuria associates with decreased extrarenal manifestations of AAV. We here provide evidence that proteinuria correlated with less involvement of sinus and joint in AAV. These observations suggested that proteinuria reflected specific renal involvement in AAV rather that systemic disease activity. This interesting phenomenon needs further investigation and has independently been observed [49].

The main limitations of our study are its retrospective design in a single center, a selection bias towards more severe cases of ANCA GN with limited information of kidney function before disease manifestation as a tertiary referral center and lack of long-term renal survival rates. Nevertheless, the number of patients with ANCA GN and severe deterioration of kidney function at our center is considerable.

## 5. Conclusions

In conclusion, proteinuria correlated with distinct clinicopathological characteristics in ANCA GN, mostly attributed to a reduced fraction of normal glomeruli. Furthermore, proteinuria in ANCA GN reflected specific renal involvement in AAV rather than systemic disease activity. Therefore, urinary findings could further improve our understanding of mechanisms promoting kidney injury and progression of ANCA GN.

## Figures and Tables

**Table 1:** Total patient cohort of ANCA GN.

Parameter	Value
Clinical data	
Female sex—no. (%)	23 (43.4)
Age (IQR)—years	65 (54.5–74.5)
Disease onset—days before admission (IQR)	18 (7–46)
Kidney biopsy—days after admission (IQR)	6 (3–9.5)
MPO/PR3 subtype—no. (%)	26/27 (49.1/50.9)
MPA/GPA subtype—no. (%)	26/27 (49.1/50.9)
New diagnosis—no. (%)	45 (84.9)
Systemic disease activity	
BVAS (IQR)—points	18 (15–20.5)
Extrarenal manifestation—no. (%)	44 (83)
Lung involvement—no. (%)	31 (58.5)
Alveolar hemorrhage—no. (%)	7 (13.2)
Sinus involvement—no. (%)	9 (17)
Joint involvement—no. (%)	12 (22.6)
Ear involvement—no. (%)	4 (7.5)
Eye involvement—no. (%)	3 (5.7)
Peripheral nerve involvement—no. (%)	6 (11.3)
Skin involvement—no. (%)	8 (15.1)
CRP (IQR)—mg/L	57.4 (19.1–107)
Renal injury	
Serum creatinine (IQR)—μmol/L	269 (116–437)
eGFR (IQR)—mL/min/1.73 m^2^	19 (9.7–50.2)
Dialysis within 30 days after admission—no. (%)	16 (30.2)
Proteinuric findings	
Leukocyturia (IQR)—per HPF	2 (1–4)
Hematuria (IQR)—per HPF	4 (3–4)
Acanthocytes—no. (%)	8 (15.1)
uPCR (IQR)—mg/g	904 (505–1653)
uACR (IQR)—mg/g	445 (164–855)
IgG/creatinine (IQR)—mg/g	44.1 (20.5–191)
α_1_-microglobulin/creatinine (IQR)—mg/g	69.6 (34.8–172)
α_2_-macroglobulin/creatinine (IQR)—mg/g	5.05 (2.92–11.1)
Histopathological findings	
Total glomeruli (IQR)—no.	17 (11–28)
Normal glomeruli (IQR)—%	48.9 (26.2–73)
Glomerular necrosis (IQR)—%	15.2 (0–44.7)
Glomerular crescents (IQR)—%	30.8 (9.76–55.1)
Glomerular sclerosis (IQR)—%	5.13 (0–26.2)
IF/TA (IQR)—%	20 (10–40)

Median values and IQR are shown. Hematuria per HPF: negative = 0, 2–4 = 1, 5–9 = 2, 10–20 = 3, >20 = 4. Abbreviations: ANCA, antineutrophil cytoplasmic antibodies; ARRS, ANCA renal risk score; BVAS, Birmingham Vasculitis Activity Score; CRP, C-reactive protein; eGFR, estimated glomerular filtration rate (CKD-EPI); GN, glomerulonephritis; HPF, high-power field; IF/TA, interstitial fibrosis/tubular atrophy; IgG, immunoglobulin G; IQR, interquartile range; MPO, myeloperoxidase; No., number; PR3, proteinase 3; uACR, urinary albumin-to-creatinine ratio; uPCR, urinary protein-to-creatinine ratio.

**Table 2:** Proteinuric findings in association with histopathological subgrouping in ANCA GN.

Berden Classification	Paratemeter	*p* Value
	uPCR	
Crescentic class—mg/g	1348 (755–1939)	
Focal class—mg/g	573 (359–1213)	
Mixed class—mg/g	1540 (729–2536)	
Sclerotic class—mg/g	5318 (5285–8129)	0.0006
	uACR	
Crescentic class—mg/g	678 (338–1047)	
Focal class—mg/g	202 (86.1–504)	
Mixed class—mg/g	1021 (458–1701)	
Sclerotic class—mg/g	3604 (3043–4429)	0.0002
	Urinary IgG	
Crescentic class—mg/g	89.1 (43.2–232)	
Focal class—mg/g	25 (11.2–96.4)	
Mixed class—mg/g	56.5 (20.1–149)	
Sclerotic class—mg/g	352 (271–542)	0.0018
	Urinary α_1_-microglobulin	
Crescentic class—mg/g	101 (52.9–197)	
Focal class—mg/g	41.4 (17.6–113)	
Mixed class—mg/g	67.2 (7.24–150)	
Sclerotic class—mg/g	191 (62.2–310)	0.1092
	Urinary α_2_-macroglobulin	
Crescentic class—mg/g	5.44 (3.55–9.03)	
Focal class—mg/g	3.13 (1.99–6.27)	
Mixed class—mg/g	10 (4.91–14.1)	
Sclerotic class—mg/g	32.8 (16.8–60.8)	0.0026

Median values and IQR are shown. Nonparametric Kruskal–Wallis test was applied for group comparisons. Abbreviations: ANCA, antineutrophil cytoplasmic antibodies; GN, glomerulonephritis; IgG, immunoglobulin G; IQR, interquartile range; uACR, urinary albumin-to-creatinine ratio; uPCR, urinary protein-to-creatinine ratio.

**Table 3:** Proteinuric findings in association with ARRS in ANCA GN.

ARRS	Parameter	*p* Value
	uPCR	
High risk—mg/g	5264 (1959–7426)	
Medium risk—mg/g	977 (627–1743)	
Low risk—mg/g	573 (359–1201)	<0.0001
	uACR	
High risk—mg/g	2946 (996–4223)	
Medium risk—mg/g	470/314–839)	
Low risk—mg/g	227 (86.1–504)	0.0003
	Urinary IgG	
High risk—mg/g	261 (246–495)	
Medium risk—mg/g	57.9 (30.8–159)	
Low risk—mg/g	24.4 (13.3–37.3)	<0.0001
	Urinary α_1_-microglobulin	
High risk—mg/g	174 (116–280)	
Medium risk—mg/g	92.3 (47.1–204)	
Low risk—mg/g	37.1 (6.24–82.9)	0.0009
	Urinary α_2_-macroglobulin	
High risk—mg/g	15.7 (8.29–28.2)	
Medium risk—mg/g	5.06 (3.06–11.1)	
Low risk—mg/g	3.33 (2.09–6.27)	0.001

Median values and IQR are shown. Nonparametric Kruskal–Wallis test was applied for group comparisons. Abbreviations: ANCA, antineutrophil cytoplasmic antibodies; ARRS, ANCA renal risk score; GN, glomerulonephritis; IgG, immunoglobulin G; IQR, interquartile range; uACR, urinary albumin-to-creatinine ratio; uPCR, urinary protein-to-creatinine ratio.

**Table 4:** Multiple regression analysis for variables associated with proteinuria.

Variable	β	SE	*p* Value
	uPCR		
Normal glomeruli—%	−47.963	14.352	0.0016
Glomerular crescents—%	−18.551	12.725	0.1513
IF/TA—%	11.895	14.609	0.4195
	uACR		
Normal glomeruli—%	−33.080	9.736	0.0014
Glomerular crescents—%	−10.111	8.633	0.2472
IF/TA—%	0.456	0.911	0.9635
	Urinary IgG		
Normal glomeruli—%	−2.237	1.269	0.0845
Glomerular necrosis—%	−1.662	1.117	0.1435
Glomerular crescents—%	0.865	1.512	0.5697
IF/TA—%	2.032	1.117	0.1129
	Urinary α_1_-microglobulin		
Normal glomeruli—%	−0.255	1.207	0.8336
Glomerular crescents—%	0.757	1.217	0.5367
	Urinary α_2_-macroglobulin		
Normal glomeruli—%	−0.333	0.073	<0.0001
Glomerular crescents—%	−0.211	0.065	0.002
IF/TA—%	−0.083	0.075	0.2726

Abbreviations: ANCA, antineutrophil cytoplasmic antibodies; GN, glomerulonephritis; IgG, immunoglobulin G; SE, standard error; uACR, urinary albumin-to-creatinine ratio; uPCR, urinary protein-to-creatinine ratio.

## Data Availability

Deidentified data are available on reasonable request from the corresponding author.
